# Poly(sodium acrylate)-Modified Magnetite Nanoparticles for Separation of Heavy Metals from Aqueous Solutions

**DOI:** 10.3390/ma15196562

**Published:** 2022-09-21

**Authors:** Magdalena Bobik, Irena Korus, Karol Synoradzki, Jacek Wojnarowicz, Dorota Biniaś, Włodzimierz Biniaś

**Affiliations:** 1 Department of Water and Wastewater Engineering, Silesian University of Technology, Konarskiego 18, 44-100 Gliwice, Poland; 2 Institute of Molecular Physics, Polish Academy of Sciences, Mariana Smoluchowskiego 17, 60-179 Poznań, Poland; 3 Laboratory of Nanostructures, Institute of High Pressure Physics, Polish Academy of Sciences, Sokolowska 29/37, 01-142 Warsaw, Poland; 4 Faculty of Materials, Civil and Environmental Engineering, University of Bielsko-Biala, Willowa 2, 43-309 Bielsko-Biala, Poland

**Keywords:** magnetic iron oxides, poly(sodium acrylate), nanoparticles, adsorption, heavy metals

## Abstract

Two types of magnetite nanoparticles: unmodified (Fe_3_O_4_ NPs), and modified with poly(sodium acrylate) (Fe_3_O_4_/PSA NPs) were synthesized by the co-precipitation method and characterized using different techniques: X-ray diffraction (XRD), transmission electron microscopy (TEM), nanoparticle tracking analysis (NTA), Brunauer–Emmett–Teller (BET) adsorption, Fourier-transform infrared spectroscopy (FTIR). Additionally, magnetic properties and the effect of pH on the zeta potential were analyzed for both types of nanoparticles. Magnetites were used as adsorbents for seven heavy metal ions (Zn(II), Cu(II), Ni(II), Cd(II), Pb(II), Cr(III), Cr(VI)) within the pH range of 3–7. Research revealed nanometric particle sizes, a specific surface area of 140–145 m^2^/g, and superparamagnetic properties of both tested materials. Moreover, the presence of PSA functional groups in modified magnetite was confirmed, which lowered the pH of the isoelectric point. Both types of magnetite were effective metal ion adsorbents, with metal cations more effectively removed on Fe_3_O_4_/PSA NPs and Cr(VI) anions on Fe_3_O_4_ NPs. The adsorption of most of the examined cations (performed at pH = 5) can be well described by the Langmuir isotherm model, whereas the adsorption of Cr(VI) ions on modified magnetite correlated better with the Freundlich model. The Dubinin–Radushkevich model confirmed that chemisorption is the predominant process. The adsorption of all metal ions was well-characterized by the pseudo-second-order kinetic model.

## 1. Introduction

With the gradual growth of the human population and its development, water contamination is becoming an increasingly significant global problem, and the provision of access to pure water sources is one of the most important criteria for proper societal functioning. From among numerous types of water contamination, particular attention is drawn to heavy metals, which count as one of the most dangerous and harmful kinds of contamination of the water environment. Although heavy metals are native components of the earth’s crust, the majority of their sources are of anthropogenic origin, and they reach water most frequently with insufficiently treated wastewater. Nowadays, various technologies are in use to eliminate heavy metal ions from water and industrial effluents. Such processes may be mentioned here as coagulation/flocculation, ion exchange, flotation, chemical precipitation, membrane processes, electrochemical treatment, or adsorption [1]. Among the aforementioned methods, the adsorption process is regarded as one of the most efficient and economical technologies. Much attention has been devoted to the development of efficient sorbents such as activated carbon, carbon nanotubes, chitosan, zeolites, polymers, functionalized silica, biosorbents, low-cost adsorbents, clay minerals, and iron oxides [2,3,4].

Many different types of iron oxides are known, including hematite, goethite, magnetite, and maghemitu [5]. They can be obtained from natural sources or synthesized in laboratory conditions. Iron oxides, as nanosize magnetic particles, are particularly attractive as adsorbents for contaminants such as heavy metal ions or dyes [4,5,6]. Their surface area is highly developed, and their magnetic properties facilitate their removal from the system after the sorption process. These advantages make magnetic iron oxides widely used in water and wastewater technologies, not only as adsorbents but also, for example, as photocatalysts [7].

Various types and forms of magnetic iron oxides have been reported as effective heavy metal adsorbents. Some examples are superparamagnetic maghemite nanoparticles for adsorption of Pb(II) and Cu(II) from aqueous solution [8], maghemite nanotubes for removal of Cu(II), Zn(II), and Pb(II) from water [9], magnetite nanorods for separation of Fe(II), Pb(II), Zn(II), Ni(II), Cd(II), and Cu(II) [10], magnetite–hematite nanoparticles for adsorption of Pb(II), Cd(II), and Cr(III) [11], mesoporous magnetite nanoparticles for simultaneous separation of Pb(II), Cd(II), Cu(II), and Ni(II) from contaminated river water [12], or magnetic iron oxide nanoparticles for competitive adsorption of As(III), As(V), and Cr(VI) [13].

An additional advantage of magnetic iron oxide adsorbents is the possibility to modify their properties through the use of various substances. Due to modifiers, it is possible to improve the stability of the nanoparticles in the dispersion, reduce their size, prevent their aggregation, and ensure an optimum surface charge or provide them with additional chemical properties [6,14]. Many materials are applied to functionalize magnetic nanoadsorbents, including inorganic materials (silica, non-metals, metals, metal oxides), carbon materials such as activated carbon or graphene oxide, and various organic compounds [4]. The latter introduces metal ion-binding functional groups such as –COOH, –OH, –NH_2_, –SH into the structure of modified materials [14]. A number of such modified adsorbents have been reported, among them, carboxylated iron oxide nanoparticles, prepared by grafting citric acid onto magnetite, used for competitive adsorption of Cd(II), Cu(II), Pb(II), and Ni(II) [15], or magnetic nanoparticles functionalized with EDTA, applied for separation of Cu(II) from aqueous solution [16]. Polymers are another group of compounds used to modify magnetic iron oxides. 3-aminopropyltriethoxysilane and copolymers of acrylic acid and crotonic acid were used to modify Fe_3_O_4_ nanoparticles, and the synthesized adsorbent was effective toward Cd(II), Zn(II), Pb(II), and Cu(II) ions [17]. Chitosan/magnetite composites enabled efficient separation of Co(II), Ni(II), and Pb(II) ions from aqueous solutions [18]. Magnetic nanoparticles covalently functionalized with polyethyleneimine were proposed for the separation of Cr(VI) anions from model solutions and industrial wastewater [19]. Magnetite modified with poly(acrylic acid) was used for adsorption of Cd(II), Pb(II), Ni(II), and Cu(II) cations [20]. The three-step modification was applied, with the final solution polymerization of acrylic acid onto synthesized nanomaterials.

This paper presents a simple method of magnetite nanoparticle modification using poly(sodium acrylate)—PSA. This polymer is well soluble in water, and the dissociated carboxyl groups in its structure are capable of binding heavy metal ions. PSA has proven to be effective against heavy metal ions, e.g., enhancing their membrane separation in the ultrafiltration method [21]. The objective of this work was to examine the possibility of the modification itself and to determine its impact on the separation capacity of the formed adsorbent toward several heavy metal ions. These aims were achieved by performing physicochemical tests (XRD, TEM, NTA, BET, FTIR, magnetic properties) of the sorbents and by making attempts to separate heavy metal ions with the use of unmodified (Fe_3_O_4_ NPs) and modified (Fe_3_O_4_/PSA NPs) magnetite at different pH values. In addition, an attempt was made to correlate the achieved separation effectiveness with the impact of pH on the adsorbents’ zeta potential in the aqueous suspension. In the final stage of the work, the adsorption isotherms and kinetics were investigated for both types of adsorbents, in an attempt to match the achieved results with the known adsorption and kinetic models.

## 2. Materials and Methods

### 2.1. Chemicals

Magnetite nanoparticles were synthesized using FeCl_3_·6H_2_O, FeSO_4_·7H_2_O, and NH_3_·H_2_O 25% solution (Stanlab, Lublin, Poland). The solution of the modifying substance at 1% concentration was prepared with the use of poly(sodium acrylate) (M_w_ = 30,000) in the form of a 40% aqueous solution (Sigma Aldrich, St. Louis, MI, USA). Model solutions of heavy metals were prepared using their inorganic salts Zn(NO_3_)_2_·9H_2_O, Cu(NO_3_)_2_·3H_2_O, Cd(NO_3_)_2_·4H_2_O, Ni(NO_3_)_2_·6H_2_O, Pb(NO_3_)_2_, Cr(NO_3_)_3_·9H_2_O, (Avantor Performance Materials Poland S.A., Gliwice, Poland), and K_2_Cr_2_O_7_ (Merck, Darmstadt, Germany). Diluted solutions of NaOH and HNO_3_ (Avantor Performance Materials Poland S.A., Gliwice, Poland) were used for pH adjustment. The concentrated 65% solution of HNO_3_ (Avantor Performance Materials Poland S.A., Gliwice, Poland) was applied for the stabilization of samples before metal content analysis. The reagents were used without additional purification.

### 2.2. Sorbent Synthesis

Magnetite nanoparticles were prepared by the Massart method [22], which involves precipitation from a mixture of iron(III) and iron(II) salts with an alkali. Based on the modified methodology employed in the paper by Liu et al. [23], weighed amounts of iron salts Fe_2_SO_4_·7H_2_O (4.2 g) and FeCl_3_·6H_2_O (6.1 g) were dissolved in 100 cm^3^ of deionized water and heated in the water bath to the temperature of 303 K. Then, during the mechanical stirring of the solution, 20 cm^3^ of ammonia solution (25%) was rapidly added and, depending on the type of the prepared material, 50 cm^3^ of deionized water or a 1% solution of poly(sodium acrylate). Thus, the prepared mixture was stirred for another 30 min. The excess unreacted reagents were washed from the precipitated magnetite by rinsing it several times with distilled water. To facilitate the precipitate sedimentation, the neodymium magnet was employed during the rinsing, whereby the magnetic properties of the sorbent were exploited. An appropriate quantity of deionized water was poured onto the prepared magnetite precipitate, forming a suspension with a known amount of dry matter of circa 10 g/dm^3^.

### 2.3. Characterization of the Adsorbent

#### 2.3.1. XRD Analysis

The quality of the obtained materials was checked by powder X-ray diffraction (XRD). Measurements were performed on an X’pert Pro (PanAnalytical, Almelo, The Netherlands) diffractometer using Cu-Kα radiation. Diffraction pattern where taken at room temperature (RT) in the range of 2*θ* = 10°–90° with the scanning rate of 0.05 s^−1^. The standard Sherrer equation was used to estimate the average crystalline size:

(1) $$D_{x}=\frac{K\cdot \lambda }{B\cdot cos\theta }$$

where *D_x_*—average crystalline size, nm; *K* = 0.9—Sherrer constant, *λ*—X-ray wavelength, nm; *B*—full width at half maximum, deg.; *θ*—XRD peak position, deg.

#### 2.3.2. TEM and NTA Analyzes

A transmission electron microscope (TEM Tecnai G2 X-Twin, FEI Company, Hillsboro, OR, USA, voltage 200 kV) was used to characterize both synthesized adsorbents (unmodified and modified with the use of PSA).

Nanoparticle tracking analysis (NTA) was used to analyze magnetite suspensions and assess agglomeration of nanoparticles. Water suspensions of Fe_3_O_4_ NPs and Fe_3_O_4_/PSA NPs were analyzed using NanoSight NS500 (light source: 405 nm (violet), Malvern Panalytical Ltd., Malvern, United Kingdom) with NTA 2.3 analytical software (version number: MAN0520-01-EN-00 (P0560F), Malvern Panalytical Ltd., Malvern, United Kingdom) to evaluate average sizes of agglomerates in the suspensions. Suspension samples were diluted with deionized water (specific conductance 0.06 S/cm) before NTA analysis. Measurements were made at a temperature of 296.5 K.

#### 2.3.3. Specific Surface Area Measurement

The specific surface area (*SSA*) of both types of magnetite was determined by means of an analysis of nitrogen adsorption isotherm with the use of the Brunauer–Emmett–Teller (BET) equation, in accordance with ISO 9277:2010. Specific surface area measurements were carried out using the Gemini 2360 v2.01 analyzer by Micromeritics Instrument Corporation (Norcross, GA, USA).

Before the measurements, vacuum desorption of the samples was performed (3.3 × 10^−2^ bar, VacPrev 061 desorption station, Micromeritics Instrument Corporation, (Norcross, GA, USA). The sample of Fe_3_O_4_ NPs was desorbed for 3 h at the temperature of 378 K. The sample of Fe_3_O_4_/PSA NPs, in turn, was desorbed for 24 h at the temperature of 303 K.

Based on the results of SSA and the theoretical density of Fe_3_O_4_ (5.17 g/cm^3^) the approximate sizes of the obtained NPs were calculated [24,25]:

(2) $$D=\frac{N\cdot 1000}{SSA\cdot \rho }$$

where: *D*—average diameter of nanoparticles, nm; *N*—shape coefficient (*N* = 6 for the spherical shape), *ρ*—sample density, g/cm^3^; *SSA*—specific surface area, m^2^/g.

#### 2.3.4. FTIR Measurement

The FTIR technique was used for analyzing the chemical structure of unmodified magnetite, poly(sodium acrylate)-modified magnetite, and the polymer itself, based on the changes of oscillations of the characteristic units of the reagents. Spectra were registered with the use of the Nicolet 6700 spectrophotometer (Thermo Fisher Scientific, Waltham, MA, USA) equipped with an “Easy-Diff” adapter. Samples in the form of a powder were placed in the recesses of measurement cells without additional mechanical working. A metal-plated mirror was the measurement background. The following measurement conditions were applied: spectral resolution: 4 cm^−1^, measurement range: 4000–500 cm^−1^, number of scans: 64.

#### 2.3.5. Magnetic Measurements

The magnetic properties were measured using a vibrating sample magnetometer (VSM), which is an option of the Physical Property Measurement System PPMS-9 (Quantum Design, San Diego, CA, USA). The magnetization as a function of temperature, *M*(*T*), was measured over a temperature range between 2 and 400 K using zero-field-cooled (ZFC) and field-cooled (FC) protocols. The magnetization as a function of the external magnetic field, *M*(µ_0_*H*), was measured at 4 and 300 K in a magnetic field up to 9 T.

#### 2.3.6. Zeta Potential Measurement

Zeta potential measurements of aqueous suspensions of samples (deionized water) were carried out using the Laser Doppler Electrophoresis (LDE) method at a temperature of 296 K (Zetasizer Nano-ZS ZEN 3600 analyzer, Malvern Panalytical Ltd., Malvern, United Kingdom). The pH of the suspensions was changed with 0.5 M NaOH and 0.5 M HCl using the automatic titration system (MPT-2 titrator, pH electrode type MV 114-S.C. SEN 0106, Malvern Panalytical Ltd., Malvern, United Kingdom; vacuum degasser, P/N 0001-6353, Systec). To determine the isoelectric point (IEP), the pH of the suspensions was modified within the ranges of 3–10 (Fe_3_O_4_ NPs sample) and 2.8–10 (Fe_3_O_4_/PSA NPs sample). For pH correction, diluted solutions of acid and base were used. They were prepared with the use of NaOH, HCl (Chempur, Piekary Śląskie, Poland), and deionized water with specific conductance below 0.1 µS/cm (HLP 20UV, Hydrolab Sp. z o.o., Straszyn, Poland).

### 2.4. Batch Adsorption Tests

To investigate the impact of pH on heavy metal ions separation, the prepared suspension of adsorbent (Fe_3_O_4_ NPs or Fe_3_O_4_/PSA NPs) was dispensed to sorption containers at the quantity of circa 5 cm^3^ (such that the quantity of the sorbent’s dry matter in each container was 50 mg). Subsequently, 20 cm^3^ of the solution of a respective metal (50 mg/dm^3^) was added to each container, and the pH was adjusted (inoLab Level 3 system, Sen Tix81 pH-electrode, Xylem Analytics Germany Sales GmbH & Co. KG, WTW, Weilheim, Germany) to the appropriate value (3, 4, 5, 6, or 7) using NaOH or HNO_3_ solution. Afterward, sorption containers were shaken for 8 h using orbital shaker SK-O330-Pro (Chemland, Stargard, Poland) at 260 rpm and 295 K. After that period, 10 cm^3^ of the supernatant were collected using the neodymium magnet in order to sediment the sorbent. Measurements of metal concentrations in the initial solutions and the supernatants were performed using the SpectrAA 880 atomic absorption spectrometer (Varian) with atomization in acetylene-air flame. All sorption experiments were made in triplicate.

The process efficiency and the adsorbent capacity were calculated using Equations (3) and (4), respectively:

(3) $$\eta =\frac{C_{i}-C_{f}}{C_{i}}$$

where *η* is the adsorption efficiency, *C_i_* and *C_f_* are, respectively, the initial and the final metal ions concentrations, mg/dm^3^;

(4) $$q_{e}=\frac{C_{i}-C_{e}}{m}\cdot V$$

where *q_e_* is the magnetite adsorption capacity at equilibrium, mg/g; *C_e_* is the concentration of metal ions at equilibrium, mg/dm^3^; *m* is the adsorbent sample weight, g; *V* is the volume of metal solution, dm^3^.

The procedure of the adsorption isotherms investigation was similar to the previous one, but the amount of adsorbent used in the sorption was equal to 25 mg, the pH was maintained at 5 and the concentration of heavy metals varied from 1 mg/dm^3^ to 70 mg/dm^3^ for almost all ions. In some cases, higher metal concentrations were used: up to 100 mg/dm^3^ (Cu(II), Cd(II) with Fe_3_O_4_/PSA NPs, Pb(II) with Fe_3_O_4_ NPs), and up to 300 mg/dm^3^ (Pb(II) with Fe_3_O_4_/PSA NPs).

Two well-known isothermal adsorption models were applied to describe the achieved results—the Langmuir model and the Freundlich model. In addition, the Dubinin–Radushkevich isotherm model was used to assess the adsorption mechanism.

The Langmuir model assumes monolayer adsorption and equal binding energy of the adsorbate particles at all binding sites of the adsorbent. The Langmuir isotherm in the linear form is represented by the following equation [26,27]:

(5) $$\frac{C_{e}}{q_{e}}=\frac{1}{q_{max}\cdot b}+\frac{C_{e}}{q_{max}}$$

where *q*_*max*_ is the maximum amount of metal adsorbed, mg/g; *b* is the Langmuir constant, dm^3^/mg.

The assumption of the Freundlich model is multilayer adsorption and heterogeneous surface (binding energy) of the adsorbent. The linear form of the Freundlich equation is given as [26,27]:

(6) $$logq_{e}=logK_{f}+\frac{1}{n}logC_{e}$$

where *K_f_* (mg/g)(dm^3^/mg)^1/n^ and *n* are experimental constants associated with the adsorption capacity and heterogeneity of the adsorption system, respectively.

The Dubinin–Radushkevich adsorption model (D-R isotherm) makes it possible to determine the average adsorption energy and assess the mechanism of adsorption (physical or chemical adsorption) from the value of this energy. The D-R isotherm in the linear form can be written as [16,26,27,28]:

(7) $$lnq_{e}=lnq_{max}-\beta \varepsilon^{2}$$

where *β*—the constant associated with the adsorption energy (mol^2^/J^2^), *ε*—the Polanyi potential (J/mol), given as:

(8) $$\varepsilon =RTln\left(1+\frac{1}{C_{e}}\right)$$

where *R*—is the universal gas constant, *T*—the temperature (K), *C_e_*—the equilibrium concentration in the solution (mol/dm^3^).

From the following Equation (9), the average adsorption energy *E* (kJ/mol) can be determined as [16,27,28]:

(9) $$E=\frac{1}{\sqrt{2\beta }}$$

The Dubinin–Radushkevich isotherm is often used to distinguish between physical and chemical adsorption mechanisms based on the *E* value. It is assumed that the *E* value < 8 kJ/mol suggests physical adsorption, while *E* values exceeding 8 kJ/mol can be attributed to chemical or ion exchange adsorption.

Similar adsorption tests were conducted to investigate the adsorption kinetics. The process parameters were as follows: 50 mg of the adsorbent, 20 cm^3^ of heavy metal solution (50 mg/dm^3^), pH of 5, and the contact time in the range of 2–240 min. The experimental data were evaluated using the pseudo-first-order and the pseudo-second-order kinetic models, which can be written as [9,29,30,31]:

- The pseudo-first-order model:
  (10) $$ln\left(q_{e}-q_{t}\right)=lnq_{e}-k_{I}t$$
- The pseudo-second-order model:
  (11) $$\frac{t}{q_{t}}=\frac{1}{k_{II}q_{e}^{2}}+\frac{t}{q_{e}}$$
  where *t*—time, min; *q_t_*—adsorption capacity at time *t*, mg/g; *k_I_*—the pseudo-first-order kinetic constant, 1/min; *k_II_*—the pseudo-second-order kinetic constant, g/(mg min).

## 3. Results and Discussion

### 3.1. Characteristics of Magnetite Adsorbents

#### 3.1.1. XRD Analysis

**Figure 1:**
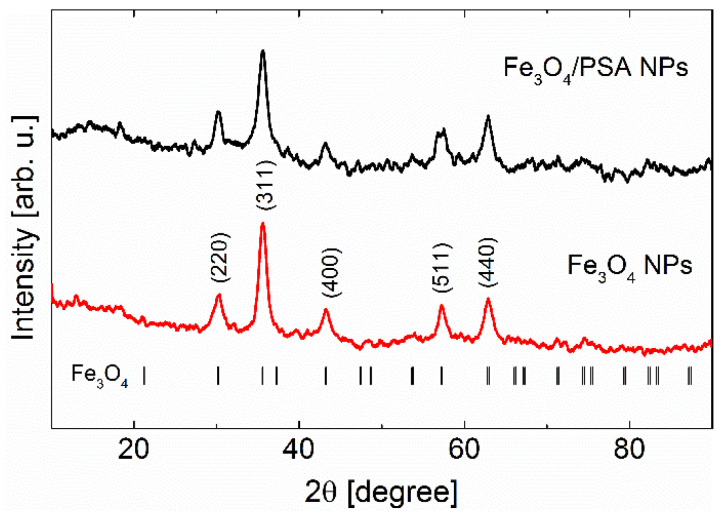
X-ray diffraction patterns of Fe_3_O_4_/PSA NPs and Fe_3_O_4_ NPs samples. The ticks at the bottom indicate the positions of the Bragg reflections for Fe_3_O_4_ cubic phase. The most pronounced peaks were described by Miller indices.

The XRD patterns of Fe_3_O_4_ NPs and Fe_3_O_4_/PSA NPs samples, shown in Figure 1, reveal diffraction peaks corresponding to the Fe_3_O_4_, which crystallizes in spinel Al_2_MgO_4_-type structure (space group *Fd*-3*m*, no. 227) [32]. At the bottom of the graph (Figure 1), the location of the Bragg peaks for magnetite (Fe_3_O_4_) structure was presented. XRD patterns registered for Fe_3_O_4_ NPs and Fe_3_O_4_/PSA NPs revealed five peaks corresponding to (220), (311), (400), (511), and (440), which are characteristic of Fe_3_O_4_. The observed peaks are broad, which may be related to the small size of crystallites. Using the standard Sherrer Equation (1), the average crystalline size for studied materials was estimated to be 7.3 ± 0.7 nm and 7.7 ± 0.8 nm for Fe_3_O_4_ NPs and Fe_3_O_4_/PSA NPs samples, respectively. The obtained values correspond with those obtained through other methods (specific surface area results and TEM). Lattice parameters obtained from the Rietveld method for both samples are equal to 8.342 ± 0.005 Å and 8.356 ± 0.007 Å for Fe_3_O_4_ NPs and Fe_3_O_4_/PSA NPs samples, respectively. Those values are consistent with the literature data [32].

#### 3.1.2. TEM and NTA Analyses

**Figure 2:**
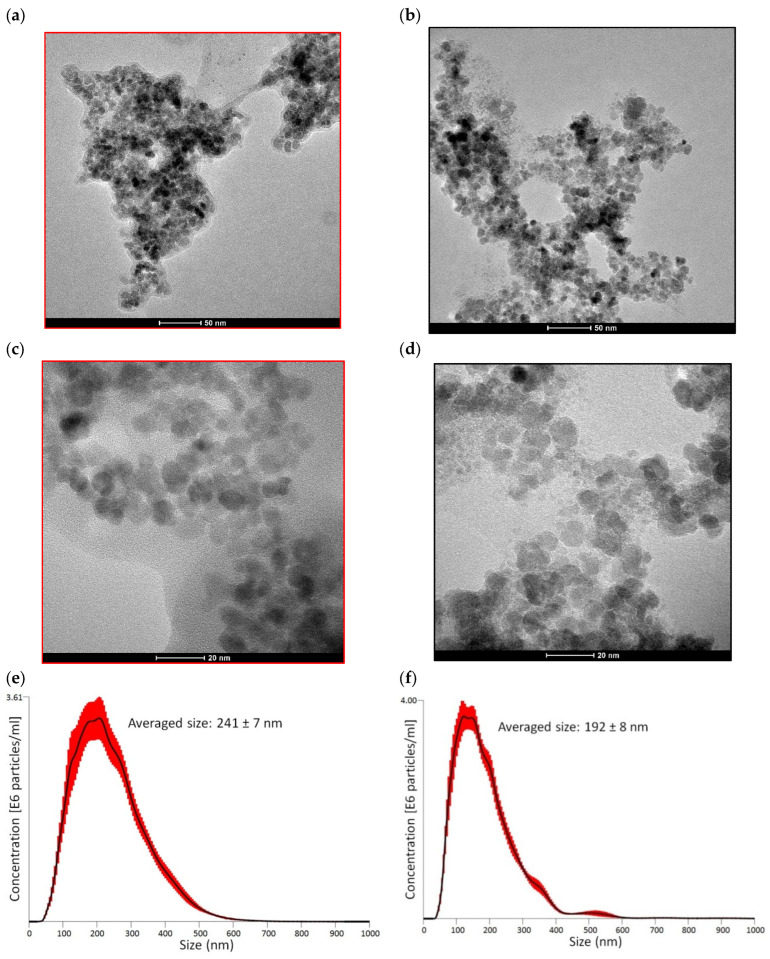
TEM images and particle size distributions by NTA method of Fe_3_O_4_ NPs (**a**,**c**,**e**) and Fe_3_O_4_/PSA NPs (**b**,**d**,**f**).

The TEM images recorded for Fe_3_O_4_ NPs and Fe_3_O_4_/PSA NPs (Figure 2a–d) show the spherical shape and nanometric size of magnetite particles in agglomerates. The particle size in both analyzed materials was similar and was estimated as 7.9 ± 1.4 nm and 7.7 ± 1.5 nm for Fe_3_O_4_ NPs and Fe_3_O_4_/PSA NPs, respectively. These values are consistent with the ones estimated based on the Formula (2) resulting from the specific surface area measurement. Similar or slightly larger sizes of synthesized Fe_3_O_4_ nanoparticles were also found by other authors [33,34,35].

One of the disadvantages of using iron oxide-based nanoadsorbents is their ability to agglomerate. Obtaining the stability of Fe_3_O_4_ NPs suspension is quite a complex issue. The kinetics of the agglomeration process of Fe_3_O_4_ NPs is dependent on such factors as pH, ionic strength values, and the content of ion types. One of the main ways to prevent/reduce NPs agglomeration is surface modification (coating) using polymers. The polymer coating obtained on the surface of the nanoparticles reduces the tendency to aggregate by providing repulsive forces between particles. It is also important to keep in mind that modifying the surface of NPs with polymers can have the opposite effect and significantly accelerate the agglomeration of NPs as confirmed by the study of Golas et al. [36]. The results of the average particle size of the suspension samples confirmed the effectiveness of surface modification in reducing the agglomeration process of Fe_3_O_4_ NPs (Figure 2e,f). The average particle size for the Fe_3_O_4_ NPs suspension sample was 241 ± 7 nm while that of Fe_3_O_4_/PSA NPs was 192 ± 8 nm. Similar values for unmodified magnetite were reported by other authors [37], and the decrease of agglomerate size after sodium citrate modification was detected using a dynamic light scattering method. Similar results were also obtained by Golas et al. [36]. In their work, they confirmed that modification of NPs with poly(sodium acrylate) can significantly reduce the agglomeration process of magnetite NPs.

#### 3.1.3. Specific Surface Area

The specific surface area (*SSA*) was calculated based on the BET isotherm equation, and the result was 139.7 m^2^/g and 144.6 m^2^/g for the Fe_3_O_4_ NPs sorbent and the Fe_3_O_4_/PSA NPs sorbent, respectively. The specific surface area of the PSA-modified magnetite was greater by 4.9 m^2^/g (3.5%) than the unmodified sample. Assuming that all particles were identical and spherical, the average sizes of Fe_3_O_4_ NPs and Fe_3_O_4_/PSA NPs calculated with the usage of the Equation (2) were 8.3 nm and 8.0 nm, respectively.

#### 3.1.4. FTIR Analysis

**Figure 3:**
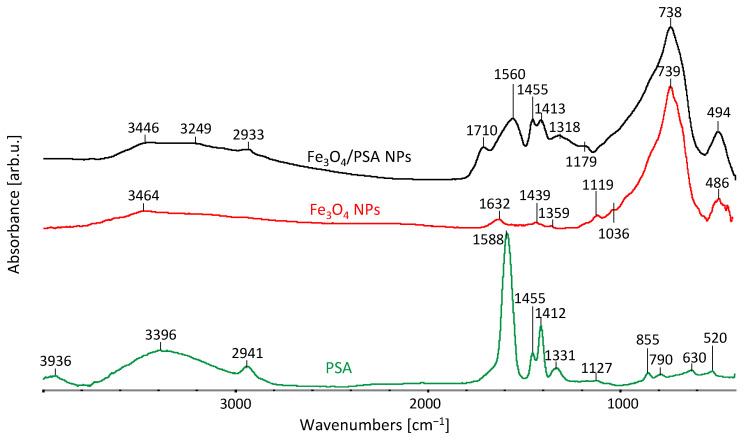
FTIR spectra of polymer (PSA), unmodified magnetite (Fe_3_O_4_ NPs), poly(sodium acrylate)-modified magnetite (Fe_3_O_4_/PSA NPs).

Figure 3 presents the spectra of unmodified magnetite (Fe_3_O_4_ NPs), poly(sodium acrylate)-modified magnetite (Fe_3_O_4_/PSA NPs) and poly(sodium acrylate) (PSA) itself.

Within the range of 3700–2600 cm^−1^, a low-intensity, broad band, related to the occurrence of stretching vibrations of hydroxyl groups (free –OH groups and hydrogen bonds) occurs in all analyzed spectra. Differences in the intensity of bands result from the differing shares of –OH groups in the hydrogen bonds [38]. This band is most clear in the PSA spectrum due to its capability to absorb water. With the wavenumber of 2941 cm^−1^ in the case of the PSA spectrum and of 2933 cm^−1^ in the case of the Fe_3_O_4_/PSA NPs spectrum, a band of symmetrical and asymmetrical stretching vibrations of ν-C-H emerges, which coincides with the band of hydroxyl group vibrations. With the wavenumber of 1710 cm^−1^, a band of stretching vibrations of carbonyl group ν_s_-C=O occurs only in the case of Fe_3_O_4_/PSA NPs [38,39]. Subsequently, a band of asymmetrical stretching vibrations of carboxyl anion ν_as_–COO^−^, characteristic of the polymer, is visible. This band’s maximum is with the wavenumber values of 1588 cm^−1^ in the PSA spectrum and of 1560 cm^−1^ in the Fe_3_O_4_/PSA NPs spectrum. The band’s shift toward a lower wavenumber may prove an interaction of carboxyl groups of the polymer with the iron ions contained in magnetite. Subsequently, at 1455 cm^−1^, a band of scissoring vibrations of δ-(CH_2_) occurs in the case of both PSA and Fe_3_O_4_/PSA NPs. At 1412 (1413) cm^−1^, a band of symmetric stretching vibrations of carboxylate anion ν_s_–COO^−^ is visible. A change in the proportion of the bands is noticeable here. It may be caused by a change in the spatial arrangement of the polymer in Fe_3_O_4_/PSA NPs, which may be a further proof of the interactions of magnetite with poly(sodium acrylate). With the wavenumber of 1331 cm^−1^ in the PSA spectrum and of 1318 cm^−1^ in the Fe_3_O_4_/PSA NPs spectrum, banding vibrations of C–H are manifested.

Bands of vibrations connected with the Fe-O oscillator in the iron oxide emerge with the wavenumber of 739 cm^−1^ and 494 cm^−1^ in the case of the Fe_3_O_4_ NPs spectrum and of 739 cm^−1^ and 486 cm^−1^ in terms of the Fe_3_O_4_/PSA NPs spectrum. In papers by other authors, bands of Fe-O group vibrations occur as a rule within the wavenumber range of 500–600 cm^−1^ [40,41], the shift of the strongest band toward higher wavenumbers can be nevertheless caused by an interaction of the residue of –OH groups, which can result from the method of preparing the sample for the FTIR analysis—drying at a relatively low temperature of 313 K. FTIR tests confirm strong interactions between magnetite and the modifier, and, in particular, a modification of the adsorbent with PSA.

#### 3.1.5. Magnetic Measurements

**Figure 4:**
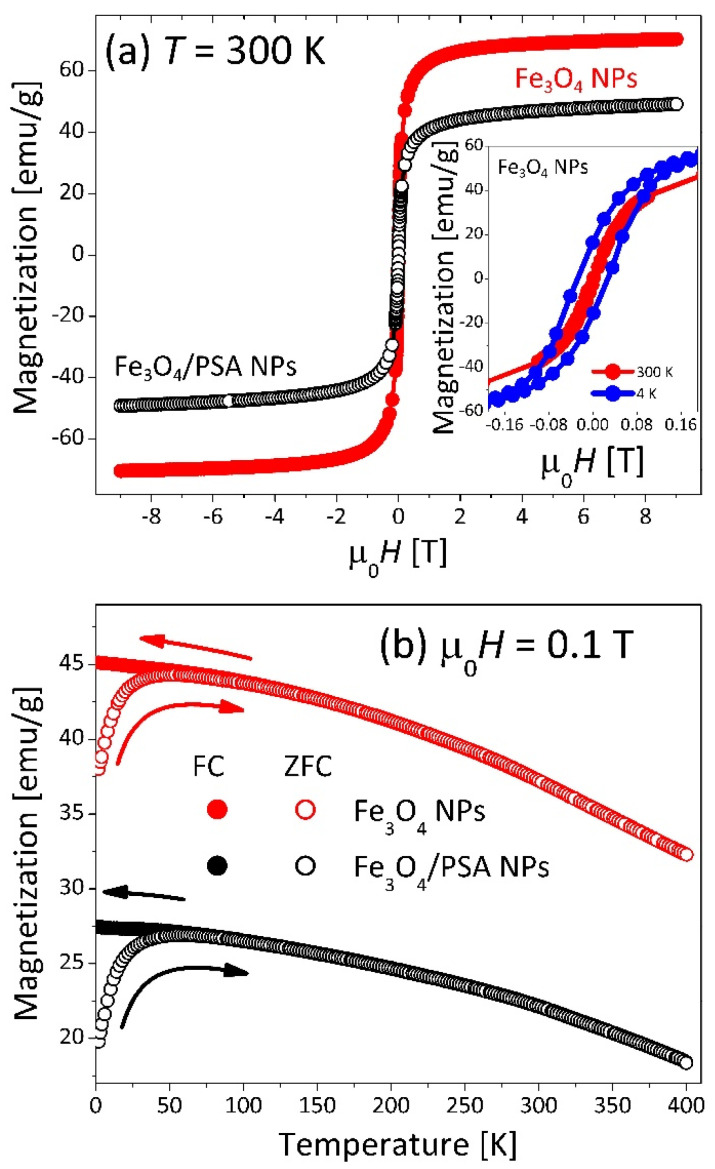
Magnetic properties of studied materials. (**a**) Magnetic field variation of the magnetization *M*(µ_0_*H*) in studied materials measured at room temperature. The inset shows a zoom-in to the low field region for magnetization curves collected at room temperature and 4 K for Fe_3_O_4_ NPs. (**b**) Temperature dependencies of the magnetization *M*(*T*) for prepared samples measured in a magnetic field of 0.1 T upon cooling the sample in zero-field (ZFC) and applied field (FC).

The magnetic properties of the synthesized magnetite adsorbents are presented in Figure 4.

Magnetization curves, *M*(µ_0_*H*), collected at room temperatures (*T* = 300 K) (see Figure 4a) showed no remanent magnetization or coercivity. In contrast, a clear hysteresis loop (inset in Figure 4a) was recorded at low temperature (*T* = 4 K). The saturation magnetization, *M*_S_, (at 300 K and 5 T) for Fe_3_O_4_ NPs is 70 emu/g, which is lower than for bulk magnetite (92 emu/g [41,42]) and higher than for bulk maghemite (γ-Fe_2_O_3_) (56 emu/g [43]). The obtained *M*_S_ value is lower than the characteristic value for magnetite by about 24% but is similar to the values given by other authors for magnetite samples synthesized from Fe(II) and Fe(III) salts using the co-precipitation method: e.g., 76 emu/g [43], 67 emu/g [11], 65.33 emu/g [33]. Lowering the value of magnetic saturation of samples synthesized, in relation to the value characteristic for the basic phase of magnetite, authors most often explain by the small particle size, significant surface development, and the possibility of partial oxidation of surface magnetite groups [11,33,41,43]. For the modified sample Fe_3_O_4_/PSA NPs saturation magnetization is even lower (49 emu/g) as it contains less magnetite. A similar decrease in magnetic saturation resulting from the modification of magnetite with molecules of other chemical substances was also observed by other authors [42,44,45,46]. The shape of the temperature dependence of the magnetization curve *M*(*T*) (Figure 4b) indicates that the magnetite particles are below 20 nm in diameter and have a spherical shape [47]. These observations are consistent with the TEM data. A distinct broad maximum in the ZFC curve observed at ~50 K manifests thermal blocking of magnetic particles. To determine the blocking temperatures, *T*_B_ ~ 10 K, we calculated the temperature derivative of the ZFC-FC difference (d(*M*_ZFC_-*M*_FC_)/d*T*) [48]. The results of magnetic measurements confirm the superparamagnetic properties of our products and are very similar to those obtained for other systems based on nanoparticles of magnetite [45,46,49,50,51].

### 3.2. Adsorption Experiments

#### 3.2.1. The Effect of pH on Sorption Effectiveness and Zeta Potential of Magnetite

**Figure 5:**
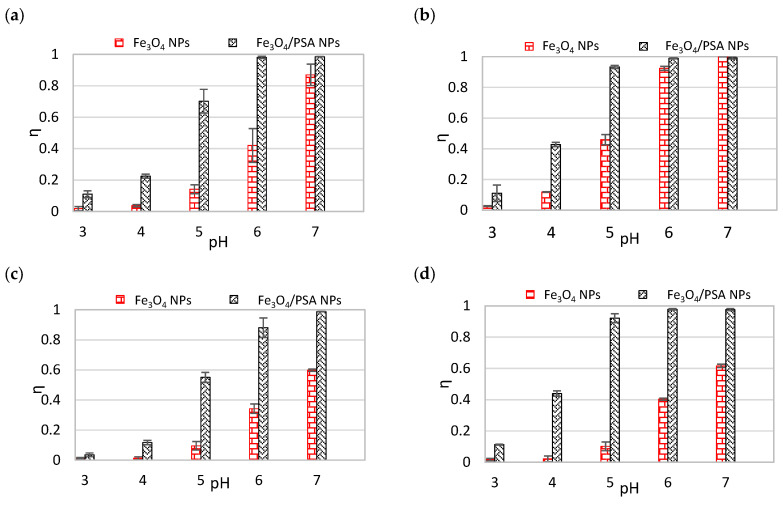

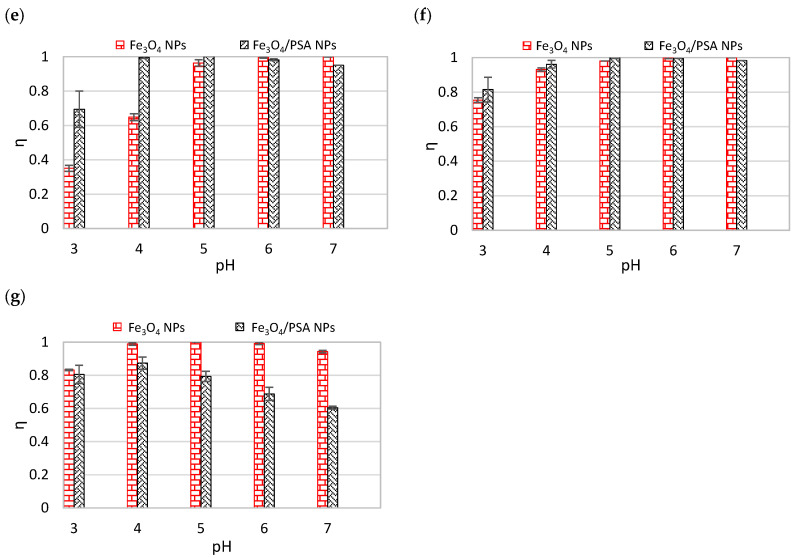
Effectiveness of unmodified magnetite (Fe_3_O_4_ NPs) and modified magnetite (Fe_3_O_4_/PSA NPs), depending on the pH, toward individual heavy metals. (**a**) Zn(II), (**b**) Cu(II), (**c**) Ni(II), (**d**) Cd(II), (**e**) Pb(II), (**f**) Cr(III), (**g**) Cr(VI).

Figure 5 presents the impact of the pH on the effectiveness of separation of individual heavy metal ions with the use of unmodified magnetite (Fe_3_O_4_ NPs) and PSA-modified magnetite (Fe_3_O_4_/PSA NPs).

When analyzing the charts presented in Figure 5, some general trends are noticeable. For the majority of the discussed heavy metals, an increase in the separation effectiveness is in line with the pH. Moreover, most of the analyzed ions (except for Cr(III) and Cr(VI)) are removed better by a sorbent synthesized with the addition of poly(sodium acrylate). The greatest differences were achieved in the case of Cd(II)—at pH = 5. The effectiveness of separation of this ion in the case of Fe_3_O_4_/PSA NPs exceeded 90%, while with the use of Fe_3_O_4_ NPs the achieved level of metal removal was only slightly above 10%. Furthermore, it can be seen that even with the highest pH value applied (pH = 7), the separation effectiveness using Fe_3_O_4_ NPs is significantly lower than 100%. A similar trend with only slight differences can be noticed in the charts for Zn(II), Ni(II), and Cu(II). Concerning Zn(II) and Ni(II) ions, the removal level that is close to 100% takes place at pH ≥ 6 in the case of Fe_3_O_4_/PSA NPs, while the maximum effectiveness of separation of those metals in the case of Fe_3_O_4_ NPs is circa 60% and over 80–90% for Ni(II) and Zn(II), respectively. When it comes to Cu(II), it is also better removed by Fe_3_O_4_/PSA NPs with pH ranging from 3 to 6, while with pH above 6, the 100% removal of this ion was accomplished with the use of both sorbents. The effectiveness of separation of Cr(III) and Pb(II) is slightly different. In the case of Pb(II), differences in sorption in favor of Fe_3_O_4_/PSA NPs are visible up to pH = 5, while above that value both sorbents display the same high level of effectiveness. Regarding Cr(III), sorption is similar in the case of both sorbents, only at the lowest tested pH values, ranging from 3 to 4, very small differences in favor of Fe_3_O_4_ NPs can be noticed. However, with pH ≥ 4, the ion removal level is almost 100% both by Fe_3_O_4_/PSA NPs and Fe_3_O_4_ NPs. In turn, Cr(VI) ions behave in a completely different manner. The observed removal level of Cr(VI) within the pH range of 3–4 is nearly 100% when using Fe_3_O_4_ NPs and circa 90% for Fe_3_O_4_/PSA NPs. With a further increase in the pH, the separation capacity of Fe_3_O_4_ NPs is maintained at a similar level of nearly 100%, while concerning Fe_3_O_4_/PSA NPs, the effectiveness of Cr(VI) adsorption gradually drops to as low as circa 60%.

The ion behavior can be explained first by the form of their occurrence in aqueous solutions and second by the interactions between them and the sorbent surface. After its introduction to the aqueous solution, magnetite is subject to hydration and its surface is fully covered with –OH groups. In a water environment, iron ions present on the oxide surface are bonded with hydroxyl groups. Surface ⁞~Fe–OH groups of hydrated iron oxide are amphoteric, therefore they can undergo acid-base dissociation. They can react with ions of dissolved acids and bases, which results in the formation of positive (–OH_2_^+^) or negative (–O^−^) charges on their surface:

$$\begin{array}{l} ⁞\sim \mathrm{Fe}–\mathrm{OH}+H^{+}\;⇌\;⁞\sim \mathrm{Fe}–{{\mathrm{OH}}_{2}}^{+}\\ ⁞\sim \mathrm{Fe}–\mathrm{OH}+{\mathrm{OH}}^{-}\;⇌\;⁞\sim \mathrm{Fe}–O^{-}+H_{2}O \end{array}$$

These protolytic reactions depend on the pH of the electrolyte solution and the surface charge of the adsorbent (its sign and magnitude) depends on the concentration of OH^−^ and H^+^ potential-forming ions [52].

One of the ways to evaluate the charge at the sorbent surface is the measurement of the zeta potential. This characterizes the charge of the broadened part of the double electric layer at the slipping plane. It is determined based on the electrophoretic mobility of the particles, which is subsequently converted into the potential value. The characteristic point with zeta potential equal to 0 is the isoelectric point (IEP). The quantity of positive and negative charges occurring at the slipping plane of the double electric layer is equal at this point. In the case of Fe_3_O_4_, the positive surface charge occurs with the value of pH < pH_IEP_, while the negative one—with the value of pH > pH_IEP_.

**Figure 6:**
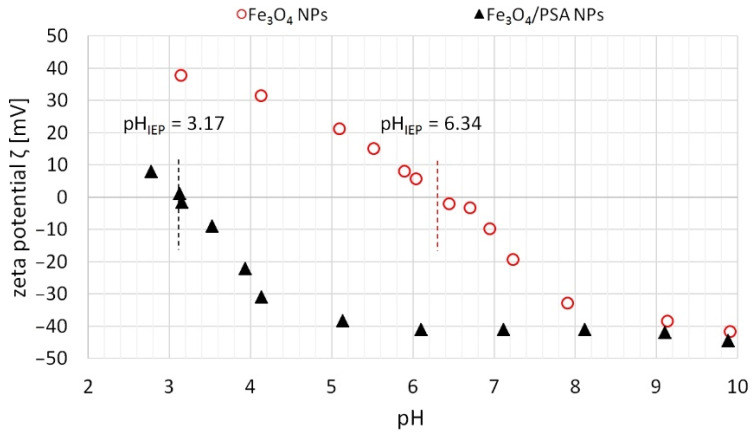
Impact of the pH on the zeta potential of unmodified magnetite (Fe_3_O_4_ NPs) and PSA-modified magnetite (Fe_3_O_4_/PSA NPs).

Figure 6 presents the impact of the pH on the zeta potential of unmodified and modified magnetite in aqueous suspension. Differences in the zeta potential behavior of the two analyzed sorbents, depending on the suspension’s pH, are obvious straight away.

When analyzing the charts, it can be noticed that up to pH 6.34 on the surface of Fe_3_O_4_ NPs positive charge prevails, but above that, the charge’s sign becomes negative. However, in the case of Fe_3_O_4_/PSA NPs, the change of the charge occurs with a far lower pH value of 3.17. The achieved pH_IEP_ = 6.34 for the Fe_3_O_4_ NPs sample is similar to the value known from the literature—5.7–6.8 [53]. As was expected, the modification of Fe_3_O_4_ NPs with the polymer caused a decrease in the pH_IEP_ value of the obtained sample, which proves a change/modification of the magnetite surface.

The visible effect of pH on the zeta potential is associated with the protonation and deprotonation of the magnetite surface during the aforementioned protolytic reactions of hydroxyl groups. This is a good explanation for the behavior of ions during their separation. With the pH below the pH_IEP_, the prevailing charge on the sorbent surface is positive, hence the prevailing phenomenon is the electrostatic attraction of anions with simultaneous repulsion of cations. In line with the increase in the pH and by exceeding the isoelectric point, the sorbent gains the resultant negative charge and the attraction of positive ions increases. However, it should be emphasized that places with a positive, negative and neutral charge can co-occur on the magnetite surface. ⁞~Fe–OH_2_^+^ groups prevail below the isoelectric point and although the overall charge is positive, ⁞~FeO^−^ groups are still present. As the pH increases, the number of ⁞~FeO^−^ groups increases, and they begin prevailing above the isoelectric point. This means that magnetite can simultaneously adsorb, to a greater or smaller extent, both positively and negatively charged compounds. In other words, when the pH increases, the equilibrium shifts such that the quantity of places in the deprotonated form increases and the zeta potential slowly assumes a negative value, which leads to an increasingly effective cation separation. Similarly, with the increase in the zeta potential, i.e., the decrease of the pH, the quantity of protonated places increases, which results in better sorption of anions [29].

Within the discussed pH range, Zn(II), Cu(II), Ni(II), Cd(II), Pb(II), and Cr(III) ions occur in the cationic form, while Cr(VI) in the anionic form. As observed, for most of the cations, better separation in the pH range analyzed was obtained using Fe_3_O_4_/PSA NPs. This is because the isoelectric point of the modified magnetite is shifted toward a lower pH (the resultant charge of the adsorbent in the solution with a pH higher than 3.17 is negative), which causes greater electrostatic attraction of the cations and the simultaneous repulsion of anions. For ions in the anionic form, in turn, Fe_3_O_4_ NPs proved more effective within the discussed pH range, which gains the resultant negative charge only at pH > 6.34, as was indicated (Figure 6).

The decrease of the zeta potential value in the case of Fe_3_O_4_/PSA NPs is caused, above all, by the nature of the polymer used. Poly(sodium acrylate), i.e., a sodium salt of poly(acrylic acid) with the general formula being [–CH_2_CH(COONa)–]_n_, is an anionic polymer, which has negatively charged carboxyl groups while in the dissociated form. The quantity of dissociated –COO^−^ groups, growing in line with the increase in the pH, will undoubtedly contribute to the lowering of the zeta potential and the pH_IEP_. The lowering of the zeta potential may also be caused by the shift of the slipping plane due to the presence of the polymer at the sorbent surface [54].

Subsequent adsorption experiments were conducted at pH = 5. This value is low enough that there is no danger of precipitation of metal hydroxides under experimental conditions. In addition, at this pH, the two adsorbents differ significantly in surface charge, which should be reflected in the adsorption results.

#### 3.2.2. Adsorption Isotherms

**Figure 7:**
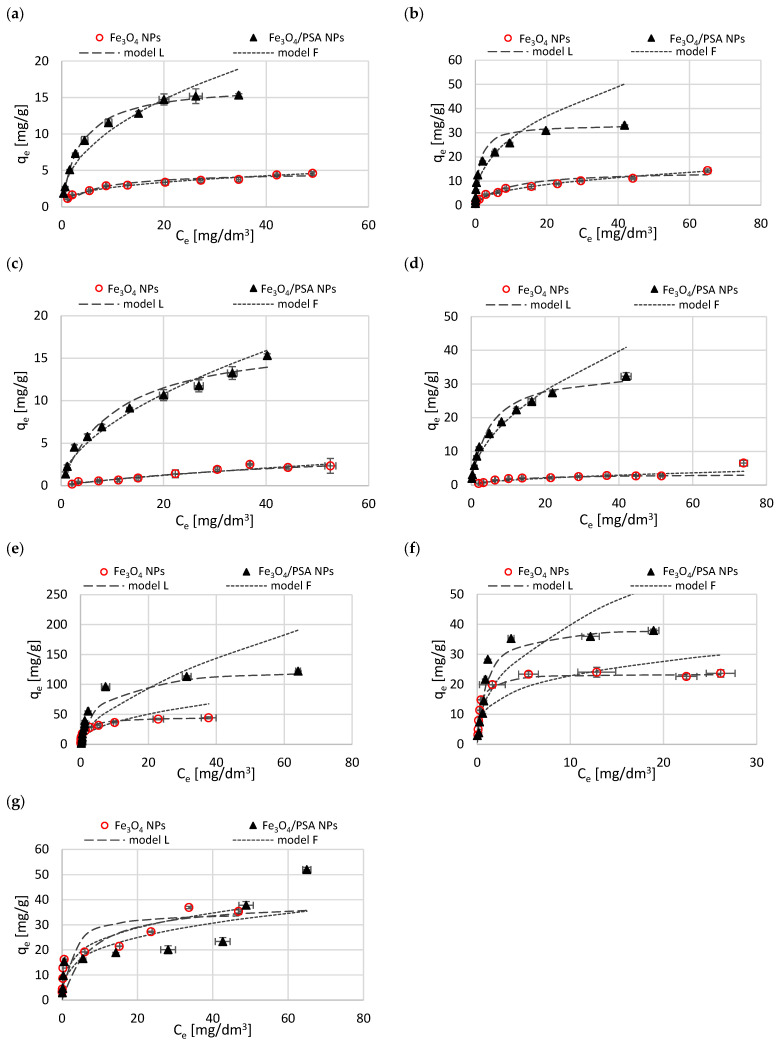
Isotherms of adsorption of Zn(II) (**a**), Cu(II) (**b**), Ni(II) (**c**), Cd(II) (**d**), Pb(II) (**e**), Cr(III) (**f**), Cr(VI) (**g**) on Fe_3_O_4_ NPs and Fe_3_O_4_/PSA NPs at pH = 5.

Adsorption isotherms determined at pH = 5 for Fe_3_O_4_ NPs and Fe_3_O_4_/PSA NPs are shown in Figure 7. The points presented in the charts constitute the averages of three experiments, and the dashed and dotted lines represent Langmuir and Freundlich models, respectively. Characteristic parameters of both models, calculated based on the linear forms of their general Equations (5) and (6), are collected in Table 1. Furthermore, Table 1 presents the mean adsorption energy *E* and the determination coefficient *R*^2^, calculated based on the Dubinin–Radushkevich isotherm model.

Through analysis of the *q_e_* = *f*(*C_e_*) graphs plotted for metal cations, it can be seen that the isotherm curves shift toward higher *q*_e_ values when changing the magnetite type from unmodified to modified with PSA. Adsorption of metal cations on the tested materials under the applied experimental conditions can be successfully characterized by the Langmuir model. Consistency between the model curves of this isotherm and the experimentally determined points, and high determination coefficients *R*^2^ (Table 1), in the range from 0.95 to over 0.99, were achieved. An exception here is the Ni(II) ion adsorbed on unmodified magnetite Fe_3_O_4_ NPs, for which a relatively low *R*^2^ value was obtained, which can be dictated by the relatively low adsorption of Ni(II) ions under these conditions. The *q*_*max*_ values, determined according to the Langmuir model, remained at a diversified level, both comparing the analyzed metal cations and different magnetite types. For all the metal cations analyzed, the polymer-modified magnetite exhibited a higher adsorption capacity than the unmodified magnetite. The highest *q*_*max*_ values were obtained for Pb(II) adsorbed on PSA-modified magnetite (129.01 mg/g). The highest (>10-fold) increase in *q*_*max*_ as a result of the replacement of unmodified magnetite with PSA-modified adsorbent was observed in the case of Cd(II) ions.

The data obtained during the adsorption of Cr(VI) on unmodified magnetite correlated well with the Langmuir model. In the case of modified magnetite, the compatibility was not so high (*R*^2^ = 0.7535), which may indicate a more complex mechanism and multilayer adsorption. However, it should be emphasized that the analysis of the same experimental data limited to the concentration of Cr(VI) 50 mg/dm^3^ showed a better correlation with the Langmuir model (*R*^2^ = 0.9221).

Generally, the use of the Freundlich isotherm model to describe the adsorption resulted in lower values of the correlation coefficient, although for most of the analyzed ions, the *R*^2^ value was satisfactorily high. In terms of two processes, adsorption of Ni(II) ions on unmodified magnetite and Cr(VI) ions on modified magnetite, the Freundlich model fitted better with the obtained data. In some other processes (adsorption of Zn(II) and Cu(II) on Fe_3_O_4_ NPs and Zn(II), Ni(II), and Cd(II) on Fe_3_O_4_/PSA NPs) the *R*^2^ coefficients determined using the Freundlich model were very high and comparable to those obtained from the Langmuir model. Many authors confirm that the adsorption of heavy metals on magnetic iron oxides and derivative adsorbents is consistent with the Langmuir model [10,29,30]. Some of them indicate that the Freundlich model can also be suitable to characterize the process [55].

The average adsorption energy *E* determined based on the Dubinin–Radushkevich isotherm model ranged from 7.77 to 15.34 kJ/mol. For most metals, the *E* value was higher than 8 kJ/mol, indicating the chemical nature of adsorption (the exception is the adsorption of Ni(II) on unmodified magnetite with E slightly lower than 8 kJ/mol). In general, *E* values were higher for PSA-modified magnetite.

#### 3.2.3. Adsorption Kinetics

**Figure 8:**
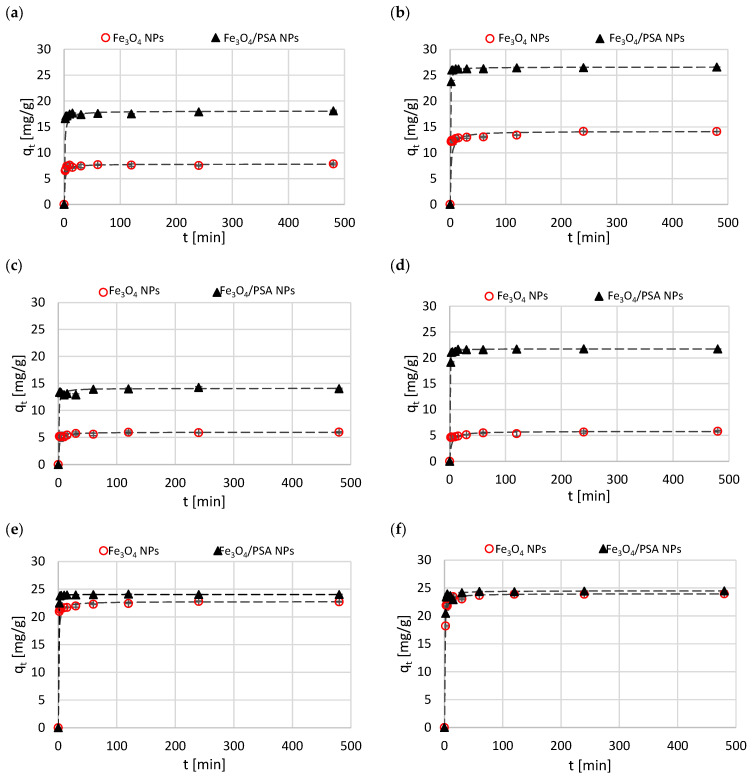

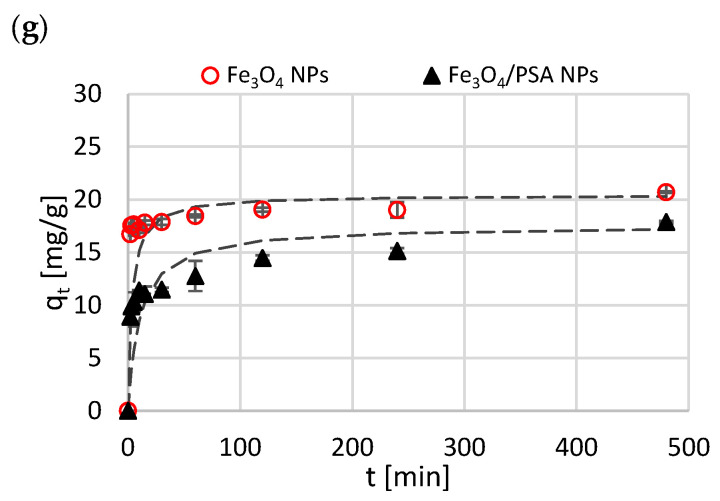
Kinetics of adsorption of Zn(II) (**a**), Cu(II) (**b**), Ni(II) (**c**), Cd(II) (**d**), Pb(II) (**e**), Cr(III) (**f**), Cr(VI) (**g**) on Fe_3_O_4_ NPs and Fe_3_O_4_/PSA NPs at pH = 5.

The impact of contact time on the process was studied for each metal ion and type of magnetite at pH = 5 (Figure 8). The points on the chart *q_t_* = *f*(*t*) present the average values from three experiments, and the pseudo-second-order kinetic model, which fits well with the experimental results, is depicted with dashed lines. The kinetic parameters for the pseudo-first- and the pseudo-second-order models are collected in Table 2.

The curves *q_t_* = *f*(*t*) indicate very rapid adsorption of metal ions in the initial stage of the process, manifested by a sharp increase in *q_t_* in the first minutes of the ions’ contact with magnetite, followed by a slowing of the process and a characteristic plateau, indicating the saturation of active sites of the adsorbent. During the first 2–3 min, adsorption of Zn(II), Cu(II), Ni(II), Cd(II), Pb(II), and Cr(III) ions, depending on the type of magnetite, exceeded 70, 80, and even 90% of the maximum value obtained in the equilibrium state. An equally high index was observed in the adsorption of Cr(VI) on unmodified magnetite (after 2 min approx. 80%), whereas adsorption of this ion on modified magnetite was slower—the amount of chromate ions adsorbed after 2 min of the process was about 50% of the maximum value.

The analysis of the collected data reveals that the pseudo-first-order kinetic model does not characterize the adsorption kinetics properly. This is evidenced by the low to moderate correlation coefficient *R*^2^ (Table 2) and the model-determined *q_e_* values that deviate from the experimentally determined amount of metal ions adsorbed at equilibrium.

In turn, the pseudo-second-order kinetic model corresponded with the results of the experiment. For all the metal-magnetite sets studied, the correlation coefficient *R*^2^ determined using this model remained in the range of 0.996–1.000 (Table 2). The values of *q_e_*, estimated from the kinetic Equation (11), fitted well with the equilibrium adsorption capacity observed during the process for individual metal ions (Figure 7).

A high level of compatibility of the experimental data with the pseudo-second-order kinetic model indicates a chemisorption as a dominant mechanism in the adsorption process [9,10,28,33,56]. However, it should be emphasized that the kinetic equations used are experimental, and the adsorption process itself is complex. Other than the chemical and/or physical binding of the adsorbate with the adsorbent, it may include other processes, e.g., diffusion from the bulk solution to the boundary layer, diffusion in the adsorbent pores, interactions between adsorbate particles, precipitation on the adsorbent surface, affecting the observed process kinetics, which makes the unambiguous assessment of the adsorption mechanism difficult.

The lack of compatibility of adsorption kinetics with the pseudo-first-order model and consistency with the pseudo-second-order rate equation is often observed in the research of adsorption kinetics of heavy metal ions on iron oxide nanoparticles (magnetite and maghemite) or their modifications with different chemical compounds. This has been confirmed in the works of other authors, among them: adsorption of Mn(II), Zn(II), Cu(II) and Pb(II) [29] and Pb(II) and Cr(VI) [33] ions on magnetite, Fe(II), Pb(II), Zn(II), Ni(II), Cd(II) and Cu(II) on magnetite nanorods [10], Cr(VI), Ni(II), Cu(II), Cd(II) and Pb(II) on magnetite-Dowex 50WX4 resin nanocomposite [28], Cd(II) and Pb(II) on sulfonated magnetic nanoparticles [30] or Cu(II), Zn(II), and Pb(II) on maghemite nanotubes [9].

## 4. Conclusions

The tests presented in this paper proved the possibility to modify magnetite nanoparticles (Fe_3_O_4_ NPs) with poly(sodium acrylate). Based on TEM and X-ray diffraction, we have shown that the magnetite particles have a spherical shape and a diameter of about 8 nm. The superparamagnetic properties of the tested materials were confirmed by magnetic measurements.

Moreover, the obtained modified sorbent (Fe_3_O_4_/PSA NPs) received new separation properties toward the analyzed heavy metal ions, with the simultaneous impact of the modification on the sorbent’s specific surface area, which was, as indicated through BET measurements, 139.7 m^2^/g and 144.6 m^2^/g for Fe_3_O_4_ NPs and Fe_3_O_4_/PSA NPs, respectively. It was proved through an FTIR test that the modified sorbent gained new functional groups coming from the polymer—above all, carboxyl groups, which contribute to a decrease in the sorbent’s zeta potential. The pH of the Fe_3_O_4_/PSA isoelectric point decreased to 3.17, while the pH of the Fe_3_O_4_ NPs isoelectric point was 6.34. Therefore, within the pH range of 3–7, in which sorption tests were carried out, modified magnetite proved to be a better sorbent for heavy metal cations, i.e., Zn(II), Cu(II), Ni(II), Cd(II), Pb(II), Cr(III), while unmodified magnetite displayed better effectiveness toward Cr(VI) ions, which occurs in the anionic form. Apart from the change of zeta potential values after the magnetite modification, the improved separation of metal cations can also be attributed to the sorbent’s acquisition of additional, anionic functional groups (–COO^−^).

For most of the metal cations tested, the adsorption process on both unmodified and PSA-modified magnetite was well-fitted with the Langmuir isotherm model, indicating monolayer adsorption. The adsorption of Cr(VI) ions on Fe_3_O_4_/PSA NPs was correlated better with the Freundlich isotherm model, which may mean that these ions adsorb in the form of multiple adsorption layers. The adsorption energy determined from the D-R isotherm indicates the chemical nature of metal ion–magnetite interactions. The adsorption process of all investigated metals was rapid and followed the pseudo-second-order kinetic model.

The easiness of modification and use and the significant improvement in the modified adsorbent’s efficiency make further work in this area desirable, e.g., the application of Fe_3_O_4_/PAS NPs for multicomponent mixtures, industrial effluents, and wastewater.

## Figures and Tables

**Table 1:** Parameters of applied model isotherms.

	Langmuir Isotherm			Freundlich Isotherm			Dubinin–Radushkevich Isotherm		
Metal Ion	*q*_*max*_ mg/g	*b* dm^3^/g	*R* ^2^	*K*_F_ (mg/g)(L/mg)^1/n^	*n*	*R* ^2^	*β* mol^2^/J^2^	*E* kJ/mol	*R* ^2^
	Fe_3_O_4_ NPs								
Zn(II)	4.83	0.1608	0.9788	1.2218	2.941	0.9777	4.30 × 10^−9^	10.79	0.6822
Cu(II)	14.24	0.1242	0.9485	2.2213	2.179	0.9696	3.40 × 10^−9^	12.13	0.9056
Ni(II)	5.26	0.0166	0.6226	0.1200	1.284	0.9473	8.27 × 10^−9^	7.77	0.8713
Cd(II)	3.34	0.0966	0.9832	0.4088	1.870	0.8880	5.35 × 10^−9^	9.66	0.8864
Pb(II)	45.71	0.5823	0.9924	12.9071	2.184	0.7435	3.28 × 10^−9^	12.35	0.7685
Cr(III)	23.65	3.3813	0.9989	12.0458	3.549	0.7720	2.25 × 10^−9^	14.89	0.7875
Cr(VI)	35.22	0.4451	0.9545	12.5142	3.581	0.9054	2.13 × 10^−9^	15.34	0.9209
	Fe_3_O_4_/PSA NPs								
Zn(II)	16.93	0.2794	0.9975	3.7484	2.187	0.9547	3.97 × 10^−9^	11.23	0.9736
Cu(II)	33.44	0.8339	0.9948	10.4807	2.375	0.9019	3.10 × 10^−9^	12.70	0.9383
Ni(II)	17.65	0.0947	0.9700	2.0014	1.774	0.9607	5.12 × 10^−9^	9.88	0.9588
Cd(II)	34.61	0.1996	0.9867	5.9572	1.939	0.9723	4.06 × 10^−9^	11.09	0.9875
Pb(II)	129.10	0.1616	0.9732	15.8655	1.665	0.7355	4.78 × 10^−9^	10.23	0.7833
Cr(III)	39.71	1.0349	0.9942	15.1007	2.326	0.8538	3.17 × 10^−9^	12.57	0.8577
Cr(VI)	39.99	0.1356	0.7535	10.0218	3.263	0.8433	2.44 × 10^−9^	14.30	0.8397

**Table 2:** Parameters of the applied kinetic models.

	1st Order			2nd Order		
Metal Ion	*q_e_* mg/g	*k_I_* min^−1^	*R* ^2^	*q_e_* mg/g	*k_II_* g/(mg·min)	*R* ^2^
	Fe_3_O_4_ NPs					
Zn(II)	0.895	0.0024	0.133	7.56	0.941	1.000
Cu(II)	2.397	0.0050	0.910	14.09	0.040	0.999
Ni(II)	0.896	0.0050	0.338	5.91	0.153	0.999
Cd(II)	1.281	0.0058	0.549	5.61	0.094	0.996
Pb(II)	2.404	0.0035	0.847	22.79	0.063	1.000
Cr(III)	2.824	0.0043	0.388	23.93	0.082	1.000
Cr(VI)	4.015	0.0026	0.659	19.07	0.058	0.999
	Fe_3_O_4_/PSA NPs					
Zn(II)	1.733	0.0020	0.469	17.84	0.117	1.000
Cu(II)	2.045	0.0019	0.219	26.48	0.200	1.000
Ni(II)	1.796	0.0042	0.731	14.27	0.060	1.000
Cd(II)	1.732	0.0025	0.273	21.75	0.205	1.000
Pb(II)	1.507	0.0013	0.160	24.06	0.830	1.000
Cr(III)	2.370	0.0034	0.346	24.47	0.106	1.000
Cr(VI)	8.032	0.0042	0.822	15.25	0.013	0.997

## Data Availability

Not applicable.
